# MCP-1-2518 (A>G) polymorphism and asthma risk: a pilot case-control study in Cameroon

**DOI:** 10.11604/pamj.2023.44.166.38544

**Published:** 2023-04-12

**Authors:** Jériel Pascal Nkeck, Jan René Nkeck, Jean-Paul Chedjou, Aude Laetitia Ndoadoumgue, Doris Bibi Essama, Emmanuel Afane Ze, Wilfred Fon Mbacham

**Affiliations:** 1 Faculty of Medicine and Biomedical Sciences, University of Yaoundé I, Yaoundé, Cameroon,; 2 Laboratory of Public Health Biotechnology, Biotechnology Centre of the University of Yaoundé I, Yaoundé, Cameroon,; 3 School of Health and Related Research, The University of Sheffield, United Kingdom,; 4 Pneumology Unit, Jamot Hospital of Yaoundé, Yaoundé, Cameroon

**Keywords:** Asthma, Cameroonians, genetics, MCP-1-2518

## Abstract

**Introduction:**

there is little data on the genetic determinants of asthma in Cameroon and sub-Saharan Africa, yet the involvement of genetics in the pathogenesis of this disease has been reported in the literature for several years. This study aims to investigate the possible role of MCP-1 2518 for the risk of asthma in Cameroonians.

**Methods:**

we performed a case-control study on 30 volunteers suffering from asthma, matched by aged and sex to 30 healthy subjects. We determine the polymorphism of MCP-1 2518 using restriction fragment length polymorphism following Polymerase Chain Reaction (RFLP-PCR). Fisher exact test was used to compare proportions, with a threshold of significance set at 0.05.

**Results:**

the average age of cases was 21±10 years with 17 (56.7%) females. The distribution of the MCP-1-2518 (A>G) gene polymorphism in people with asthma was as follows: 3 for AA, 5 for GG, and 22 for AG. The minor G allele was predominant (90%) in people with asthma. It was significantly associated with asthma whether the genotype was heterozygous AG or homozygous GG (p<0.01).

**Conclusion:**

MCP-1-2518 (A>G) shows an association with asthma in our sample. Future larger studies evaluating several polymorphisms are needed to describe the genetic determinants of asthma in Cameroon and sub-Saharan Africa.

## Introduction

Asthma is a major public health problem affecting more than 300 million people worldwide according to the Global Initiative for Asthma (GINA) [1]. It is a heterogeneous group of diseases characterized by chronic inflammation of the airways which can lead to characteristic and potentially fatal clinical manifestations. It´s a global concern, especially in developing and middle-income countries such as Cameroon [2,3]. The genetic susceptibility of asthma has been reported in several studies in literature. Suspected genes may have an effect on bronchial hyperreactivity, and/or on bronchial inflammation, which represents one of the major processes in the pathophysiology of asthma, and one of the main axis allowing the development of new therapeutics [4,5].

Inflammation means the production of inflammatory proteins such as chemokines which are small chemotactic molecules with a major role in leukocyte maturation and migration [6]. One of them, the monocyte chemo-attractant protein 1 (MCP-1), produced by fibroblasts, lymphocytes, endothelial cells, macrophages, and smooth muscle cells, belongs to the CC chemokine family, and is one of the possible biomarkers in people with asthma [7,8]. In fact, it contributes to the migration and infiltration of monocytes in inflammatory zones and acts on the selection and activation of macrophages in inflammatory processes of the upper airways [9]. The polymorphism of MCP-1-2518 (A>G) affects the promoter region of MCP-1 and its further expression leading to an impaired level of MCP-1 protein which may affect the inflammatory processes [10]. Although studies have identified an association between MCP-1-2518 (A>G) and asthma, no studies are available in Cameroonians on the genetics of asthma to confirm this potential association. The purpose of this study was to investigate the possible roles of MCP-1-2518 (A>G) and its polymorphisms in individuals with asthma living in the city of Yaoundé (Cameroon). This is the first genetic study on asthma in Cameroon.

## Methods

The methodology and results were reported according to the Strengthening the Reporting of Genetic Association studies (STREGA) statement [11].

**Study design:** we conducted a pilot study with a case-control design, from June to December 2017 in Yaoundé (Cameroon).

**Setting:** this study was carried out from January 1^st^ to August 31^st^, 2017, in three facilities. Jamot Hospital in Yaoundé and the Respiratory Disease Clinic in Nkolbisson for the recruitment of participants. Yaoundé Jamot Hospital is a 2^nd^ category hospital in the health pyramid of Cameroon; it is the first referral hospital for pneumology in the country. The analyses were performed in the Laboratory of Public Health Biotechnology of the Biotechnology Center of the University of Yaoundé I, Cameroon.

**Participants:** the cases were made of 30 volunteers with asthma consulting at the Yaoundé Jamot Hospital or the Respiratory Disease Clinic of Nkolbisson. They were recruited consecutively. The controls were 30 healthy volunteers matched to cases by sex and age for a ratio of 1: 1. Controls were recruited from families where there are no cases of asthma, in the general population of the city of Yaoundé, invited to participate through a press release. The sample size was estimated using Cochran (1965: 75) formula with 80% power and 5% error [12]. The sampling was consecutive.

**Data collection:** ethical clearance was obtained from the Institutional Ethical Review Board of the Faculty of Medicine and Biomedical Sciences of the University of Yaoundé I (Cameroon). All the participants read and signed informed consent before the inclusion. All the data were collected directly from the participants. It comprised socio-demographics data such as age, sex, ethnic group (Bantu, semi-Bantu, Sudano-Sahelian), personal and family history of asthma, comorbidities, and the severity of the disease (by the GINA 2016). After the clinical evaluation, for each participant, we collected a drop of blood further placed on a sterile filter paper and stored in an envelope containing silica gel at 25 °C in a closed box.

**Figure 1:**
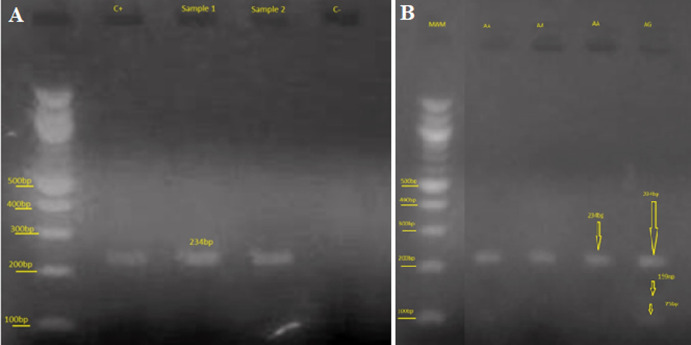
visualization of MCP-1 after amplification (A), and after digestion (B) (MWM: molecular weight marker; C+: positive control; C-: negative control; bp: bases pairs; AG and AA represents the genotypes)

**Molecular analysis:** they were carried out at the Laboratory of Public Health Biotechnology of the Biotechnology Center of the University of Yaoundé I, using RLFP-PCR (Restriction Fragment Length Polymorphism-Polymerase Chain Reaction). Primary deoxyribonucleic acid (DNA) extraction on filter paper was perfomed by chelex method; secondary DNA extracts were amplified with PCR using specific primers. Primers for each variant were formatted by the New England Biolabs. A total volume of 25 μl of Polymerase Chain Reaction (PCR) mixture containing 22 μl of the PCR master mix (with 0.25 μl of each primer), and 3 μl of DNA extracts were prepared for amplification. The following primers were used to study the polymorphism of the MCP-1-2518 (A>G): forward 5 'gene - TCT CTC ACG CCA GCA CTG ACC-3' and reverse 5'-GAG TGT TCA CAT AGG CTT CTG -3´. The PCR was performed using a T3 thermocycler designed by Biometra®. The program was set as follows: an initial pre-denaturation phase at 95°C for 3 minutes, followed by 35 cycles for denaturation at 95°C each least 45 seconds, then followed by primers´ binding step at 55°C for 45 seconds, and elongation at 72°C for 45 seconds, and a final elongation step at 72°C for 5 minutes [13]. At the end, the PCR products (8μl) were digested overnight at 37°C using 2.5U PvuII restriction endonuclease (Fermentas, Germany) for the MCP-1-2518 (A>G) gene polymorphism. The digested products were visualized after migration on a 2% agarose gel electrophoresis. A single 234 bp band was identified as AA (ancestral homozygous genotype), 159 bp, and 75 bp identified as GG (minor homozygous genotype), and 234 bp, 159 bp, and 75 bp identified as AG (heterozygous genotype) (Figure 1).

**Statistical analysis:** it was performed using Statistical Package for Social Sciences (SPSS) software version 21.0. Continuous variables were expressed in terms of mean and standard deviation. Categorical variables were expressed in terms of counts and their proportions. Proportions of alleles and genotypes were compared using Fisher´s exact test. The threshold of significance was set at 5%.

**Ethics approval and consent to participate:** all the participants read and signed an informed consent sheet. Research authorizations were obtained from the institutions involved. Ethical clearance was obtained from the Institutional Ethical Review Board of the Faculty of Medicine and Biomedical Sciences of the University of Yaoundé I (Cameroon).

## Results

**Characteristics of the sample:** forty people with asthma were approached for the study, of whom 30 agreed to participate. To these were matched 30 of 35 controls who agreed to participate in the study and were eligible. Each group of individuals consisted of 30 persons, of which 13 (43.3%) were men and 17 (46.7%) were women. The mean age was 21 ± 10 years for cases and 24 ± 6 years for controls. The predominant ethnic groups were Bantu (18/30 in cases and 17/30 in controls), followed by Semi-Bantu (8/30 in cases and 6/30 in controls). There were 9 (30%) patients with a family history of asthma. Mild persistent asthma was the most common clinical form occurring in 19 (63.3%) cases, while intermittent asthma, moderate persistent asthma, and persistent severe asthma accounted for respectively in 4 (13.3%), 2 (6.6%), and 5 (16.6%) cases.

**Polymorphism of MCP-1 gene and association with asthma:** all participants underwent genetic analysis. The minor allele G was found in 27 (90%) volunteers with asthma and 6 (20%) controls, whereas the major allele A was found in 25 (83.3%) patients and 28 (93.3%) controls. The heterozygote genotype AG was more frequent in cases (73.3%) while the homozygote AA was the most frequent in controls (88.9%) (Table 1). The allele G was significantly associated with asthma (p<0.001), but the frequencies of the allele A were not different in cases and controls (p=0.4). The genotype AA was more represented in controls (p<0.001), while AG and GG were more found in cases (p<0.001) (Table 1). In addition, we found a potential association between the MCP-1 (A>G) gene polymorphism and the severity of asthma (Table 2).

**Table 1:** genotype distribution and allele frequencies of MCP-1 2518 (A>G) polymorphism

Polymorphisms	Cases (n=30)	Controls (n=30)	p-value
**Genotypes**			
AA	3 (10%)	24 (80%)	p < 0.001
AG	22 (73.3%)	4 (13.3%)	p < 0.001
GG	5 (16.7%)	2 (6.7%)	p < 0.001
**Alleles**			
A	25 (83.3%)	28 (93.3%)	p = 0.4
G	27 (90%)	6 (20%)	p < 0.001

**Table 2:** association of MCP-1 2518 (A>G) polymorphism and asthma severity

Asthma severity	Alleles				Genotypes			
	A (n=25)	p-value	G (n=27)	p-value	AA	AG	GG	p-value
Intermittent	4 (16%)	p<0.001	1 (3.7%)	p<0.001	3 (10%)	1 (3.03%)	0 (0%)	p<0.001
Mild persistent	19 (76%)		19 (70.4%)		0 (0%)	19 (63.3%)	0 (0%)	
Moderate persistent	2 (8%)		2 (7.4%)		0 (0%)	2 (6.6%)	0 (0%)	
Persistent severe	0 (0%)		5 (18.5%)		0 (0%)	0 (0%)	5 (16.6%)	

## Discussion

Although they are necessary, genetic studies are uncommon in sub-Saharan Africa, even more in asthma where the involvement of genetics in pathogenesis is no longer in doubt. There are recognized genetic and molecular biomarkers of asthma reported in literature. Our study aimed to study the implication of the polymorphism of the MCP-1 gene in people with asthma in Yaoundé, Cameroon. The major result is that the polymorphism of the MCP-1 gene could be a determinant of asthma in our sample.

The monocyte chemo-attractant protein 1 (MCP-1) is produced by fibroblasts, lymphocytes, endothelial cells, macrophages, and smooth muscle cells, and is thought to be involved in the activation and regulation of endothelial cells and it is indirectly involved in angiogenesis by recruiting cells [14]. Since the year 1999, Rovin *et al*. have demonstrated that massive expression of the MCP-1 gene was responsible for the risk of developing severe acute asthma [15]. It contributes to the migration and infiltration of inflammatory cells especially monocytes in bronchial inflammatory zones and acts as a chemoattractant, increasing the numbers and activity of macrophages [8]. The polymorphism of MCP-1-2518 (A>G) affects the promoter region of MCP-1 and its further expression will lead to an impaired level of MCP-1 protein which may affect this inflammatory processes [16]. Chemoattractant proteins such as MCP-1 also interact not only with each other but also with helper T lymphocytes involved in the respiratory tract defense; a disturbance in these interactions due to mutations in the genes of these chemotractants may contribute to an increased risk of developing asthma and other respiratory allergies [7,8,14].

The mutation on the MCP-1-2518 A>G was found in 53.3% of cases contrary to 13.3% of controls. Similar results were found by Molfino *et al*. in 2007 and Tuder *et al*. in 2012, which respectively found frequencies of the minor G allele between 50 and 55% in people with asthma in Turkey [17,18]. The frequencies of heterozygous and homozygous phenotypes AG and GG differ with other results found in literature, like Bagci *et al*. in 2015 in Turkey, who found a frequency of 46.1% of the phenotype AG (heterozygote) and 40.4% among controls in Turkey [13]. We found MCP-1 (A>G) polymorphism may influence asthma severity. This result was similar to those found by Molfino *et al*. in 2007, Tuder *et al*. in 2012, and Szalai *et al*. in 2001 [17-19]. This suggested that not only the susceptibility to asthma, but also its severity could be determined by this gene in Cameroonians. However, some authors have rather found MCP-1 genes as a protective factor in Africans as opposed to Caucasians, however, few studies have been conducted to date to verify this assertion [20]. However, there is always a complexity of interaction between single-nucleotide polymorphisms (SNPs) that can vary within populations, hence the importance of increasing the number of studies in order to get a clearer picture [21].

The potential implications of the association between the MCP-1 gene polymorphism and asthma are primarily preventive. Based on this gene, it will be possible to better identify predisposed subjects and to set up strategies to limit the impact of the environment on the onset of asthma, and to detect asthma at the preclinical stage. At the diagnostic level, it will be possible to use the MCP-1 protein assay as a biomarker of asthma and its severity [22,23]. Finally, certain therapeutic strategies are developed taking into account the inhibition of the synthesis and action of MCP-1 [24].

The interpretation of the results of this study must, however, take into account certain limitations including the small sample size, and serum levels of MCP-1 protein that were not measured to assess gene expression.

## Conclusion

The minor allele G of the MCP-1 gene is found on 90% of people with asthma in our sample, and could thus be a genetic determinant of asthma in this sample. Thus, further studies should be carried out on a large sample, including more polymorphism in people suffering from asthma.

### What is known about this topic

- *Genetic factors play a predominant role in the pathogenesis and transmission of asthma;*
- *The MCP-1 protein plays a role in respiratory inflammation, which is involved in asthma;*
- *The MCP-1 gene is associated with a higher risk of developing asthma and greater severity in certain populations*.

### What this study adds

- *Mutations in the MCP-1-2518 gene (A>G) are found in 9 out of 10 Cameroonians with asthma;*
- *The presence of the minor G allele of the MCP-1 gene appears to be significantly associated with asthma in Cameroonians;*
- *The minor G allele of the MCP-1 gene could be a potential biomarker of asthma severity in Cameroonians*.
